# Palliative care in Dutch hospitals: a rapid increase in the number of expert teams, a limited number of referrals

**DOI:** 10.1186/s12913-016-1770-2

**Published:** 2016-09-23

**Authors:** A. Brinkman-Stoppelenburg, M. Boddaert, J. Douma, A. van der Heide

**Affiliations:** 1 Department of Public Health, Erasmus Medical Center, University Medical Center Rotterdam, Room NA22-12, P.O. Box 2040, 3000 Rotterdam, CA The Netherlands; 2 Netherlands Comprehensive Cancer Organisation (IKNL), Godebaldkwartier 419, 3511DT Utrecht, The Netherlands

**Keywords:** Palliative care, Palliative medicine, Referral and consultation, Hospitals, Observational study

## Abstract

**Background:**

Palliative care expert teams in hospitals have positive effects on the quality of life and satisfaction with care of patients with advanced disease. Involvement of these teams in medical care is also associated with substantial cost savings. In the Netherlands, professional standards state that each hospital should have a palliative care team by 2017. We studied the number of hospitals that have a palliative care team and the characteristics of these teams.

**Methods:**

In April 2015, questionnaires were mailed to key palliative care professionals in all general, teaching and academic hospitals in the Netherlands. Out of 92 hospitals, 74 responded (80 %).

**Results:**

Seventy-seven percent of all participating hospitals had a palliative care team. Other services, such as outpatient clinics (22 %), palliative care inpatient units (7 %), and palliative day care facilities (4 %) were relatively scarce. The mean number of disciplines that were represented in the teams was 6,5. The most common disciplines were nurses (72 %) and nurse practitioners (54 %), physicians specialized in internal medicine (90 %) or anaesthesiology (75 %), and spiritual caregivers (65 %). In most cases, the physicians did not have labeled hours available for their work as palliative care consultant, whereas nurses and nurse practitioners did. Most teams (77 %) were only available during office hours. Twenty-six percent of the teams could not only be consulted by healthcare professionals but also by patients or relatives. The annual number of consultations for inpatients per year ranged from 2 to 680 (median: 77). On average, teams were consulted for 0.6 % of all patients admitted to the hospitals.

**Conclusion:**

The number of Dutch hospitals with a palliative care team is rapidly increasing. There are substantial differences between teams regarding the disciplines represented in the teams, the procedures and the number of consultations. The development of quality standards and adequate staffing of the teams could improve the quality and effectiveness of the teams.

**Electronic supplementary material:**

The online version of this article (doi:10.1186/s12913-016-1770-2) contains supplementary material, which is available to authorized users.

## Background

In the Netherlands, palliative care is an integral part of regular healthcare. Dutch government policy is based on the idea that palliative care is generalist care and should therefore be provided by any healthcare professionals whenever necessary. As such, palliative care is not a distinct medical specialty as it is in many other countries. Core elements of palliative care, such as basic symptom management and aligning treatment with patients’ goals, should be integrated in care as it is delivered by any healthcare professional. In case of complex problems, such as managing refractory symptoms or negotiating a difficult family meeting, specialist palliative care should be available. Palliative care teams (PCTs) can be consulted by professionals involved in palliative care and can provide such specialist palliative care, either in or outside the hospital. This model of palliative care delivery resembles the model as described by Quill and Abernethy [1] which distinguishes primary palliative care (which includes skills all clinicians should have) and specialist palliative care (which includes skills for managing more complex and difficult problems) [1]. In the Netherlands, PCTs are available throughout the country since the start of this century. Currently, 30 regional PCTs are mainly consulted by general practitioners, nursing home physicians and home care nurses, but not by hospital-based care professionals [2]. In the Netherlands, two thirds of patients with advanced incurable disease are admitted to hospital at some time during their last three months of life [3]. Of cancer patients older than 65 years, 29 % dies in hospital, a percentage that is low compared to other countries [4]. Hospital care is usually focused on diagnosis, treatment and discharge, and several studies have reported unmet needs and deficiencies in the quality of care of patients dying in the hospital [5–7]. PCTs in hospitals have been shown to have positive effects on patients’ quality of life and satisfaction with care [8–11]. In order to improve hospital palliative care, the Dutch Federation of Oncological Societies (SONCOS) has stated in their “Multidisciplinary standards for oncological care in the Netherlands” that each hospital should have a PCT by 2017 [12].

This development underlines the important role PCTs are expected to play in supporting professional caregivers and in providing specialized palliative care.

The aim of this study is to investigate the number of hospitals that currently have a PCT and to study the characteristics of these teams.

## Methods

### Study design and data collection

In April 2015 we performed a cross-sectional study. An online questionnaire was sent to key persons in palliative care in all 92 general, teaching and university hospitals in the Netherlands, including two oncology centers. The key persons were care professionals who are known to have an important role in the development of palliative care in their hospital. In case of non-response, these persons were contacted after several weeks by mail or phone to remind them of the study and to invite them to fill in the questionnaire.

### Population and setting

In total, 74 questionnaires were returned (response 80 %); responses came from general hospitals (*n* = 43), teaching hospitals (*n* = 23) university hospitals (*n* = 7) and one oncology hospital. Non-responding hospitals included both hospitals with and without PCTs.

### Questionnaire

The key persons were requested to fill out a 78 item questionnaire (see Additional file 1) which was based on a questionnaire from a former study [13]. It was pretested by two PCT members. Based upon this test, the wording of some questions was improved. After an introduction and some general questions on the provision of palliative care in their hospital, the questionnaire focused on the PCT, if applicable. Questions were asked about the disciplines that were represented in the PCT, the procedures followed by the team, the number of consultations, team meetings and quality assurance procedures.

### Statistical analysis

We analysed the data using SPSS version 20.

## Results

### Palliative care in hospitals

**Fig. 1:**
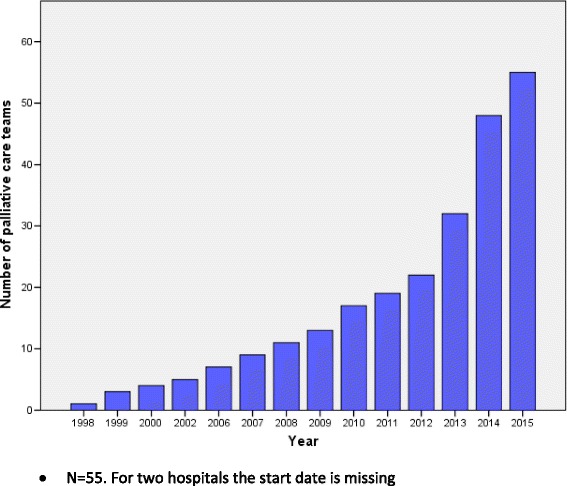
Number of hospitals with a palliative care team

Palliative care is on the agenda of most hospitals (Table 1). The majority of hospitals has an assignment from the board of directors or medical staff to develop palliative care (82 %) or has a steering committee implementing palliative care (85 %). Fifty-four percent of all hospitals have a palliative care policy of some form. Overall, 77 % of hospitals have a PCT and the other 23 % are in the process of starting one. The number of teams has increased rapidly over the last 3 years (Fig. 1). Other palliative care facilities, such as labeled palliative care beds (20 %), inpatient units for palliative care (7 %), outpatient palliative care clinics (22 %) and palliative daycare facilities (4 %) are relatively scarce. A vast majority of all hospitals use measurement instruments (91 %) to assess symptom burden. Most frequently used instruments are the Distress Thermometer (73 %), Numeric Rating Scales (NRS)/Visual Analogue Scales (VAS) (24 %) and the Utrecht Symptom Diary/Edmonton Symptom Assessment Scale (ESAS) (18 %). Respondents remarked that these instruments and guidelines are often not used throughout all hospital wards. Ninety-six percent of hospitals follow national palliative care guidelines, although respondents remarked that not every healthcare professional is familiar with these guidelines. Seventy-three percent of all hospitals have one or more wards with nurses that have palliative care as their special field of interest and education.

**Table 1:** Hospital characteristics (*N* = 74)

	Overall *N* = 74 n (%)
Number of beds	
- 0–500	47 (63)
- 501–1500	25 (34)
- Missing	2 (3)
Palliative care policy plan	40 (54)
Assignment from the board of directors or medical staff to develop palliative care	61 (82)
Palliative care steering group	63 (85)
Palliative Care Team	57 (77)
Number of PCTs that started before 2012	19 (34)
Number of PCTs that started before 2010	13 (25)
Outpatient clinic palliative care	16 (22)
Palliative daycare	3 (4)
Palliative care labeled beds	15 (20)
Labeled beds are concentrated on a unit for palliative care	5 (7)
Palliative care nurse champions	54 (73)
Use of measurement instruments	67 (91)
Use of palliative care guidelines	71 (96)
Use of care pathway for the dying	52 (70)

### Palliative care teams

#### Number of consultations

In 2014, 50 out of the 74 hospitals had a PCT. The annual number of inpatient consultations per team ranged between 2 and 680, with a median of 77.

PCTs that started before 2012 have substantially more consultations (median 160 consultations) compared to PCTs that started after 2012 (median 39 consultations). PCTs in university hospitals have more referrals compared to PCTs in general and teaching hospitals. PCTs in university hospitals tend to have started earlier compared to teaching and general hospitals. There are no other differences between different types of hospitals. All PCTs can be consulted for inpatients, but only 28 teams can also be consulted at the outpatient clinic, with a median annual number of consultations for outpatients of 20 (range 2–384). Ten teams were willing to visit patients at home with a median annual number of six home visits (range 1–74). Twenty-four percent of the respondents stated that the number of consultations exceeds their capacity; 47 % stated that the number of consultations is less than their capacity. The annual number of palliative care consultations for inpatients as a percentage of the total annual number of hospital admissions ranged between 0.01 and 2.3 %, with a mean of 0.6 %.

#### Disciplines represented in the PCT

The most common disciplines represented in the PCTs are nurses (72 %) and nurse practitioners (54 %), physicians specialized in internal medicine (90 %) or anesthesiology (75 %) and spiritual caregivers (65 %). Both psychologists and social workers participate in 28 % of the teams. In the majority of teams, nurses and nurse practitioners have labeled hours for their work as a palliative care consultant. However, the majority of medical specialists, social workers and spiritual caregivers have no labeled hours. For the minority that does, the mean number of labeled hours varies between 1 and 4 h per week. About one third of the teams include a general practitioner and another third includes a nursing home physician (see Additional file 2).

#### Procedures followed by the PCT

Table 2 describes characteristics of the procedures followed by the PCT. All teams can be consulted by medical specialists, 79 % can be consulted by nurses, 40 % by paramedics and 26 % by patients or relatives. 11 % of the PCTs is available 24/7. Most consultations involve face to face contact of the PCT with the patient (81 %). Seventy-two percent of all teams have explicit referral criteria. There are different types of transmural collaboration. In about half of the cases (54 %) the PCT consists of professionals both from inside and outside the hospital. Most teams are involved in other activities, such as palliative care education inside (95 %) and outside (51 %) the hospital, development of protocols (81 %) and scientific research (33 %). A vast majority of teams (95 %) have a weekly multidisciplinary team meeting to discuss patients that were referred to them (Table 3).

**Table 2:** Characteristics of the procedure followed by the palliative care team (*N* = 57)

	Overall *N* = 57 n (%)
The team has specified referral criteria	41 (72)
Who can consult the PCT?	
- Medical specialist	57 (100)
- Co-assistant	24 (42)
- Paramedics	23 (40)
- Nurses	45 (79)
- Patients and/or relatives	15 (26)
For which type of patients can the PCT be consulted?	
- Clinical patients	57 (100)
- Patients at the outpatient clinic	35 (62)
- Patients who are known by the PCT and who are staying at home	29 (51)
- Patients who are not known by the PCT and who are staying at home	13 (23)
Availability of the PCT	
- During office hours	51 (89)
- 24 h/ 7 days a week	6 (11)
The advice is given:	
- Mostly bedside	46 (81)
- Mostly face to face with referring professional	40 (70)
- Mostly by telephone	16 (28)
Is there a standard follow up of the patient?	
- Mostly	28 (49)
- Sometimes	26 (46)
- Never	3 (5)
Is there a standard follow up with the referring professional?	
- Mostly	30 (53)
- Sometimes	27 (47)
Is there standard deliberation with the transfer nurse about the situation at home?	
- Yes, always	4 (25)
- On indication	37 (65)
- No	6 (11)
Is there standard deliberation with the general physician – nursing home physician before discharge?	
- Yes, always	15 (26)
- On indication	30 (53)
- No	12 (21)
Members of the PCT visit patients at home	13 (23)
Forms of out-patient/in home collaboration	
- The PCT consists of professionals both form inside and outside the hospital	31 (54)
- The PCT provides consultation by telephone for patients who reside outside the hospital	19 (33)
- The PCT provides bedside consultation outside the hospital	11 (19)
- Consultants from regional PCTs’ perform bedside consultation in the hospital	5 (9)
- The PCT does not work transmural	13 (23)
Other activities of the PCT	
- Scientific research	19 (33)
- Education inside the hospital	54 (95)
- Education outside the hospital	29 (51)
- Development of protocols	46 (81)

**Table 3:** Characteristics of the palliative care consultation teams meetings (*N* = 57)

	Overall *N* = 57 n (%)
The frequency of the PCT’s meetings is at least once a week	54 (95)
Is the persons who request the consultation present at the PCT’s meeting?	
- Always/often	15 (26)
- Sometimes/seldom/never	42 (74)
Which type of patients are discussed at the meeting?	
- All patients	26 (46)
- Only complex patients	6 (11)
- Only new patients	5 (9)
- Only new and complex patients	20 (35)
A report with the PCT’s advice is sent to the general practitioner/nursing home physician	30 (53)
A report with the PCT’s advice is sent to the person who requested the consultation	43 (75)
Members of the PCT are present at multidisciplinary team meetings of other hospital departments.	33 (56)

#### Quality aspects

Most teams make use of national palliative care guidelines and measurement instruments (90 %) (Table 4). The measurement instrument used most often by the PCT is the Distress Thermometer (56 %).

**Table 4:** Quality aspects of the palliative care team (*N* = 57)

	Overall *N* = 57 n (%)
The PCT uses guidelines and measurement instruments	51 (90)
The PCT has specified quality criteria	37 (65)
The PCT has set criteria regarding the education of team members	52 (91)
There is education for the team as a whole	30 (53)
Attention is paid to ‘care for carers’^a^	35 (61)
There are team meetings for issues not concerning patient care	
- Yes, regularly	27 (46)
- Yes, incidentally	29 (51)
- No	1 (2)

^a^Care for carers refers to caring for the healthcare professionals

Sixty-five percent of the PCTs have defined quality criteria for providing their advice. The most frequently defined criterion is that the PCT advice is given within 24 h. Ninety-one percent of PCTs has specified some requirements regarding the PCT members’ expertise. However, a broad range of educational programs is mentioned and there is no consensus regarding the required education for each participating discipline.

#### Supporting and impeding factors for the development and implementation of the PCT

Respondents were asked to mention factors that either supported or impeded the development and implementation of the team. The most frequently mentioned supportive factors were enthusiasm and motivation of the PCT members, including a role as ‘ambassador’ for the team of nurses and nurse practitioners (46 %), aspects regarding functioning of the team (e.g. accessibility and availability of the team, response to referrals and educational activities) (47 %), receiving (financial) support from the hospital management (22 %) and satisfaction of patients and referring physicians who acknowledge the added value of the team (16 %). Impeding factors for successful development of a PCT are lack of finances (77 %), lack of commitment and/or financial support by the hospital management (19 %), lack of awareness regarding the existence of the PCT (18 %) and a (negative) attitude of some medical specialists and nurses towards the PCT (18 %). Some respondents (5 %) mentioned late referral to the PCT as an impeding factor.

## Discussion

The awareness of palliative care in Dutch hospitals is increasing. A vast majority of hospitals has an assignment from the board of directors or medical staff to develop palliative care or a palliative care steering committee. The percentage of hospitals with a PCT has risen from 39 % in 2013 [13] up to 77 % in 2015. In all likelihood, the norms set by the Dutch Federation of Oncological Societies (SONCOS), which state that each hospital should have a PCT by January 2017, contributed to the substantial increase in numbers of teams [12]. As positive as this increase in number of PCTs may be, the characteristics of the PCTs also show us substantial differences between the teams. Teams that started before 2012 have substantially more referrals than ‘younger’ teams. It is known that the establishment of a PCT takes time. In the literature, many barriers to consultation of PCTs have been described. These include misconceptions that palliative care is only appropriate for patients nearing death or that involving palliative care professionals can be conceived by patients as a sign that there is no hope left [14, 15]. Because of these misconceptions PCTs are often consulted late in the disease trajectory [15–19].

In studies that show positive effects of PCTs, these teams are often consulted relatively early in the a patient’s disease trajectory and often in the outpatient clinic [8, 11, 20]. While the percentage of hospitals with palliative care outpatient clinics rose from 11 % in 2013 to 22 % in 2015 [13], still less than a quarter of hospitals offer their patients this opportunity for early palliative care support.

Besides differences in the number of consultations, there are also differences in the working processes of the teams, in disciplines participating in the teams, in the expertise teams require from their members and in the availability of the team and involvement in care for out-patients. Furthermore, there is no consensus regarding the use of measurement instruments. Non-specialized care professionals in hospitals as well as PCTs use a wide variety of measurement instruments. In this survey, most commonly used instrument by both generalists and specialists in palliative care is the Distress Thermometer, an instrument originally validated as a screening tool for psychological distress, that is now also in some places used to screen patients for referral to a PCT [21, 22].

In a 1-day observational study in 14 Belgian hospitals, it was found that 9.4 % of all patients admitted to the hospital are in the palliative phase, which was defined as the phase where a patient is suffering from an incurable, progressive, life-threatening disease, without a prospect of remission, stabilization or improvement [23]. A study by Gardiner et al. in two acute hospitals in the UK showed that 36 % of all hospitalized adult patients were identified as having palliative care needs according to the Gold Standards Framework criteria (criteria that support professionals to identify patients who are nearing the end of life and to assess their needs, symptoms and preferences [24]), whereas medical staff identified 15.5 % of patients as having palliative care needs [25]. In our survey we found that the annual number of palliative care consultations as a percentage of the total annual number of hospital admissions, was 0.6 %. This is low compared to other countries, such as the United States where an average rate of 4.4 % was found [26]. This implies that more patients in hospitals could benefit from specialist palliative care.

Furthermore, the number of labeled hours that members of the PCTs have for their work as an expert palliative care consultant, is very low, especially for physicians. This is in line with a recent report of the Economic Intelligence Unit on the Quality of Death Index. Although the Netherlands are ranked 8^th^ on the overall score and 2^nd^ on palliative care and healthcare environment, the score on human resources is relatively low (22^nd^). This means that the availability of specialists in palliative care and healthcare professionals with general knowledge of palliative care is low, as is the availability of appropriate training [27]. This is confirmed by the lack of adequate financing of the PCTs in hospitals in our study.

Dumanovsky et al. conclude that higher staffing levels (full-time equivalents of PCT members per 10.000 admissions) were associated with higher service penetration (the annual number of palliative care consultations as a percentage of the total annual number of hospital admissions). In their study, palliative care programs with the highest staffing levels (≥2.7 FTE per 10.000 admissions) reached a service penetration of 6.5 %. Higher service penetration was associated with shorter time to the initial palliative care consultation [26].

### Strengths and limitations

This nationwide study demonstrates the increasing number of PCTs in the Netherlands. It demonstrates variations between the number of referrals and working procedures of the teams. The results can have implications for the development of new (models for) PCTs. A strength of this study is the relatively high response rate. Among the non-responders were both hospitals with and without PCTs. Therefore we can conclude that this study gives a good overview of current palliative care practices in Dutch hospitals.

A limitation is that our study does not give insight in the quality of palliative care in Dutch hospitals or in the quality of the PCT involvement. From a recent comparison between different countries, using data from 2010, it was found that end-of- life care in the Netherlands is characterized by a relatively low percentage of hospital deaths, a low percentage of intensive care admissions and a low use of chemotherapy in the last 180 days before death [4]. This suggests that there may be a relatively high awareness among Dutch healthcare professionals of the need to refocus care when the end of life approaches, although no firm conclusions can be drawn based on these data regarding the quality of palliative care in Dutch hospitals. We recommend further studies to monitor the development of these PCTs and to gain more insight in the timing and the quality of palliative care team involvement in Dutch hospitals.

## Conclusion

Palliative care in Dutch hospitals is often supported by PCTs. The number of these teams has rapidly increased over the last few years, but there are substantial differences between teams regarding the disciplines represented in the teams, the procedures and the number and timing of referrals. The involvement of PCTs in care for incurably ill patients is relatively limited.

To stimulate the further development and implementation of PCTs in hospitals, we recommend the development of a formalized quality framework with models for (transmural) palliative care team consultation, to improve the quality of palliative care in hospitals. Such a framework, that should be adopted by professional organizations and policymakers, can form the basis for the development of quality criteria and quality assessment of PCTs. The concepts and borders of generalist and specialist palliative care should be discussed and where possible defined, so that criteria can be set regarding the education of all disciplines involved. Furthermore, adequate staffing of the PCTs is necessary to increase the number of PCT consultations.

## Additional files

Additional file 1: Palliative care teams nationwide questionnaire survey - 2015. (DOCX 37 kb) Additional file 2: Disciplines represented in the palliative care consultation teams. (DOCX 19 kb)

## Abbreviation

PCT: Palliative care consultation team
