# Supplementary material for: Palliative care in Dutch hospitals: a rapid increase in the number of expert teams, a limited number of referrals
Source: BMC Health Serv Res. 2016 Sep 23;16:518. doi: 10.1186/s12913-016-1770-2 (PMC5035474; doi:10.1186/s12913-016-1770-2)
Supplement: Additional file 1: — Palliative care teams nationwide questionnaire survey - 2015. (DOCX 37 kb) [file 12913_2016_1770_MOESM1_ESM.docx]

**Palliative care teams nationwide questionnaire survey - 2015**

*Netherlands Comprehensive Cancer Organisation (IKNL), Utrecht, Netherlands & Erasmus MC, University Medical Center Rotterdam, Department of Public Health, Rotterdam, the Netherlands*

I. General questions regarding palliative care in hospital
1. What is the name of the hospital?
2. What is your position within the hospital?
3. Type of hospital

- General hospital
- Teaching hospital
- University hospital
- Oncological centre

4. How many inpatient beds does the hospital have?
5. How many admissions does the hospital have on an annual basis?
6. Is there a palliative care team in the hospital?

- Yes
- No

7. Is there an outpatient clinic for palliative care?

- Yes
- No

8. Is there a daycare unit for palliative care?

- Yes
- No

9. Are there labeled beds for palliative care?

- Yes
- No

10. Are the labeled beds concentrated on a designated palliative care unit?

- Yes
- No

11. Are there wards with nurses that have palliative care as their special field of interest and education?

- Yes
- No

12. Is there an assignment from the board of directors or medical staff to develop palliative care in the hospital?

- Yes
- No

13. Is there a committee or steering group for palliative care?

- Yes
- No

14. Does the hospital have a palliative care policy plan?

- Yes
- No

15. Under what organizational unit is palliative care situated?

II. Questions regarding the quality and use of tools hospital-wide

16. Are measurement instruments (e.g. The Distress Thermometer) used to assess symptom burden?

- Yes
- No

17. If so, who uses the measurement instrument (which disciplines)?

18. Does the hospital staff work according to palliative care guidelines ?

- Yes
- No

19. Does hospital staff work with the care pathway for the dying?

- Yes, on the following wards:
- No

*The following questions in this survey are about consultation of a palliative care team. Therefore the following question is asked again:*

20. Is there a palliative care team in your hospital?

- Yes
- No

III. General questions regarding the palliative care team

21. What is the name of the palliative care team?
22. When did the team start? (Month / year)
23. Is there, in addition to the palliative care team, a separate pain team?

- Yes
- No

24. If so, there is a clear division of tasks between the palliative care team and the pain team?

- Yes
- No
- Explanation:

25. What was the number of inpatient consultations for the PCT in 2014?
26. What was the number of outpatient consultations for the PCT in 2014?
27. What was the number of home visits of the PCT in 2014?

IV. Questions regarding the disciplines represented in the palliative care team.
28. Which discipline / specialty is the coordinator / leader of the team?

29. Which discipline are included in the palliative care team?

Please indicate the number of labeled hours per week for each specialty. In addition, specify the extra efforts (in hours) for the palliative care team, if applicable.

|  | Labeled hours per week: | | Average extra effort (in hours) |
| --- | --- | --- | --- |
| Anesthesiologists |  | |  |
| Chaplains / spiritual caregivers |  | |  |
| Hospice doctors |  | |  |
| General practitioner |  | |  |
| Internist (oncologists) |  | |  |
| Clinical geriatricians |  | |  |
| Pulmonologists |  | |  |
| Radiotherapists | |  |  |
| Oncology nurses | |  |  |
| Pain nurses | |  |  |
| Other nurses | |  |  |
| Nurse specialists | |  |  |
| Secretaries | |  |  |
| Nursing home physicians | |  |  |
| Psychologists | |  |  |
| Social workers | |  |  |
| Other, namely: | |  |  |

30. Which disciplines can be consulted on indication by the palliative care team?

| - Anesthesiologists |
| --- |
| - Chaplains |
| - Hospice doctors |
| - General physicians |
| - Internist (oncologists) |
| - Clinical geriatricians |
| - Pulmonologists |
| - Social workers |
| - Oncology nurses |
| - Pain nurses |
| - Other nurses |
| - Nurse specialists |
| - Psychologists |
| - Radiotherapists |
| - Secretaries |
| - Nursing home physicians |
| - Physiotherapist |
| - Psychiatrists |
| - Other, namely: |

V. Questions regarding the financing of the palliative care team

31. How is the palliative care team financed? (multiple answers possible)

- From the DOT palliative care
- Own financing from the hospital
- Otherwise, namely:

32. Is the DOT palliative care registered?

- Yes
- No

33. Is the DOT palliative care submitted to health insurance companies?

- Yes
- No

34. Are there any agreements with health insurance company about reimbursement?

- Yes
- No

35. Are there any internal agreements about the financial costs of the team with the board / medical staff?

- Yes
- No

36. Was the number of consultations in 2014 in accordance with the number of labeled hours
for the members of the palliative care team?

- No, our team actually had too many consultations
- No, our team actually had too few consultations
- Yes, the number of consultations was in line with the number of labeled hours

VI. Questions regarding procedures followed by palliative care team

37. Are there established criteria so that health care professionals know in which cases they can consult the palliative care team?

- Yes
- No

38. Who can refer patients to the palliative care team? (multiple answers possible)

- Medical specialists
- Physicians (not) in training
- Interns
- Nurses
- Paramedics
- Patient / family
- Otherwise, namely:

39. For which diagnostic group can the palliative care team be consulted?

- Only oncological patients
- All patients
- Otherwise, namely:

40. For what type of patients can the palliative care team be consulted? (multiple answers possible)

- Inpatients
- Outpatients
- Patients who stay at home (or elsewhere) and are known by the palliative care team
- Patients who stay at home (or elsewhere) and who are not necessarily known to the palliative care team
- Otherwise, namely:

41. At what times can the palliative care team be consulted?

- 24 hours / 7 days a week
- Within office hours
- Otherwise, namely:

42. How is the consultation organized outside office hours?

43. How does the request for consultation come in? (multiple answers possible)

- By phone
- Via the electronic patient record
- Via a paper form
- Via the multidisciplinary team meeting
- Otherwise, namely:

44. In what way is the consultation of the team given? (multiple answers possible)

- Usually by telephone
- Mostly bedside
- Usually face-to-face with requester
- Otherwise, namely:

45. How does the counseling take place? (multiple answers possible)

- Usually by telephone
- Usually face to face with consultation requester
- Only recorded in electronic medical record
- Only noted in paper patient files / consultation form

46. ​​Is there a standard time available for a (first) contact?

- No, this varies per patient
- Yes, namely: ... (several minutes)

47. Is the patient as a standard rule seen by one or more members of the palliative care team?

- No, usually the consultation is done by telephone
- Yes, the patient is always seen but only by the nurse
- Yes, the patient is always seen by a nurse and a physician
- Yes, the patient is always seen but only by the physician
- Otherwise, namely:

48. How is the palliative care team physicians’ position regarding the patients?

|  | Never | Seldom | Sometimes | Often | Always |
| --- | --- | --- | --- | --- | --- |
| As treating physician |  |  |  |  |  |
| As a fellow practitioner |  |  |  |  |  |
| As a consultant |  |  |  |  |  |

49. What is the average number of contacts of the team with the patient?
50. Where is the consultation registered? (multiple answers possible)

- Paper patient record
- Electronic patient record
- PRADO
- Otherwise, namely:

51. Is there standard feedback provided to the referrer?

- No
- Yes, sometimes
- Yes, usually

52. Is there standard follow up of the patient?

- No
- Yes, sometimes
- Yes, usually

53. Is there contact between the transfer nurse and the palliative care team, prior to the dismissal, regarding palliative care at home?

- Yes, always
- Only on indication
- No, because…..

54. Is there contact between the palliative care team and the nursing home physician or general practioner, prior to the dismissal, regarding palliative care at home?

- Yes, always
- Only on indication
- No, because…..

55. Does the palliative care team provide advice or counseling after the patient is dismissed from hospital? (multiple answers possible)

- No
- Yes, patients are standard called by telephone
- Yes, patients are seen at the outpatient clinic
- Yes, members of the palliative care team visit the patient, if necessary, at the place where the patient is staying
- Yes, other:

56. Do members of the palliative care team visit patients outside the hospital?

- No
- No, not yet, but there are plans to do so in the future
- Yes

57. Our transmural way of working is apparent from: (multiple answers possible)

- We do not work transmural
- Composition of the palliative care team with professionals from the hospital and the community
- Telephone consultation for patients outside the hospital
- Bedside consultation outside hospital
- Community based health care professionals perform bedside consultation in hospital
- Otherwise, namely:

58. What activities does the palliative care team perform for the purpose of publicity and profiling? (multiple answers possible)

- Research
- Training (within the hospital)
- Training (outside the hospital)
- Development of protocols
- Otherwise, namely:

VII. Questions regarding the multidisciplinary working of the palliative care team

59. How often is there a multidisciplinary team meeting (MTM) of the palliative care team ?
(.... Times per ....)

60. What is the average duration of the multidisciplinary team meeting? (In minutes)
61. Do referrers attend the MTM?

- Always
- Mostly
- Sometimes
- Rarely
- Never

62. Which disciplines attend the multidisciplinary team meetings?

|  | Always present | Sometimes present |
| --- | --- | --- |
| Anesthesiologist |  |  |
| Referrers |  |  |
| Occupational therapist |  |  |
| Physiotherapist |  |  |
| Chaplain/spiritual caregiver |  |  |
| Hospice doctor |  |  |
| General practitioner |  |  |
| Internal medicine specialist (oncologist) |  |  |
| Clinical geriatrician |  |  |
| Pulmonologist |  |  |
| Social Worker |  |  |
| Neurologist |  |  |
| Oncology nurse |  |  |
| Pain nurse |  |  |
| Psychologist |  |  |
| Psychiatrist |  |  |
| Nurse specialist |  |  |
| Psychologist |  |  |
| Radiotherapist |  |  |
| Secretary |  |  |
| Nursing home physician |  |  |
| Physiotherapist |  |  |
| Other disciplines, namely: |  |  |

63. Which patients are discussed in the MTMs?

- All patients
- Only new patients
- Only complex patients
- New and complex patients

64. How many patients are on average discussed in the MTMs?

65. Does the referrer always receive a report of the MTM?

- No
- Yes, explain

66. Does the general practitioner or nursing home physician always receive a report of the MTM?

- No
- Yes, explain

67. Is a member of the palliative care team present at MTMs in other departments?

- No
- Yes, in department (s):

VIII. Questions regarding the quality of the palliative care team

68. Does the palliative care team use of specific guidelines, protocols and instruments (eg. palliative care guidelines, the distress thermometer, ESAS, HADS)?

- No
- Yes, explain

69. If so, what instruments are used?

- Physical domain:
- Psychological / psychiatric domain:
- Social domain:
- Spiritual / existential domain:
- Cultural domain:
- Last 24 hours:
- Loss and grief:
- Legal and ethical area:

70. Are there agreed quality criteria? (E.g., the patient must be seen within 24 hours after referral)

- No
- Yes, regarding:

71. Does the palliative care team check whether the advice has been followed?

- Yes
- No

72. Are there any specific requirements regarding the expertise of members of the palliative care team? (education / training requirements)

- No
- Yes, regarding:

73. What education and / or training in the field of palliative care do members of the palliative care team receive?

- Physicians:
- Nurses:
- Paramedics:
- Spiritual caregivers:
- Other disciplines:

74. Is the palliative care team educated / trained collectively?

- No
- Yes, explain

75. Is attention paid to care for the carers in the palliative care team?
76. Does the palliative care team have non-patient-related meetings (to evaluate processes and results)?

77. What are / were impeding factors in the development and implementation of the palliative care team?
78. What are / were supporting factors in the development and implementation of the palliative care team?

Thank you for your cooperation!
