# Supplementary material for: Palliative care in Dutch hospitals: a rapid increase in the number of expert teams, a limited number of referrals
Source: BMC Health Serv Res. 2016 Sep 23;16:518. doi: 10.1186/s12913-016-1770-2 (PMC5035474; doi:10.1186/s12913-016-1770-2)
Supplement: Additional file 2: — Disciplines represented in the palliative care consultation teams. (DOCX 19 kb) [file 12913_2016_1770_MOESM2_ESM.docx]

**Disciplines represented in the palliative care consultation teams (n=57)**

|  | All  n (%) | Labeled hours per week - n |
| --- | --- | --- |
| Nurse*   - Nurses without labeled hours - Nurses with labeled hours | 41 (72) | |
|  | - 8 (20) | |
|  | - 33 (80) | 1-2 : 4 |
|  |  | 3-8: 4 |
|  |  | 9-20: 10 |
|  |  | 21-40: 7 |
|  |  | >40: 8 |
| Nurse practitioner   - Nurse practitioners without labeled hours - Nurse practitioners with labeled hours | 31 (54) | |
|  | - 6 (21) | |
|  | - 23 (78) | 1-2 : 2 |
|  |  | 3-8 : 3 |
|  |  | 9-20 : 5 |
|  |  | 21-40 : 11 |
|  |  | >40 : 2 |
| Anesthesiologist   - Anesthesiologists without labeled hours - Anesthesiologists with labeled hours | 44 (77) | |
|  | 25 (61) | |
|  | - 16 (39) | 1-2 : 11 |
|  |  | 3-8 : 4 |
|  |  | 9-20 : 1 |
|  |  | 21-40: - |
|  |  | >40 : - |
| Internal medicine specialist   - Internal medicine specialists without labeled hours - Internal medicine specialists with labeled hours | 51 (90) | |
|  | 29 (62) | |
|  | - 18 (38) | 1-2 : 7 |
|  |  | 3-8: 7 |
|  |  | 9-20: 2 |
|  |  | 21-40: 1 |
|  |  | >40: 1 |
| Radiotherapist   - Radiotherapists without labeled hours - Radiotherapists with labeled hours | 15 (26) | |
|  | 11 (85) | |
|  | - 2 (15) | 1-2 : 1 |
|  |  | 3-4: 1 |
|  |  | 9-20:- |
|  |  | 21-40:- |
|  |  | >40:- |
| Lung specialist   - Lung specialists without labeled hours - Lung specialists with labeled hours | 33 (58) | |
|  | - 22 (73) | |
|  | - 8 (27) | 1-2 : 6 |
|  |  | 3-8: 1 |
|  |  | 9-20:1 |
|  |  | 21-40:- |
|  |  | >40:- |
| Clinical geriatrician   - Clinical geriatricians without labeled hours - Clinical geriatricians with labeled hours | 27 (47) | |
|  | - 14 (63) | |
|  | - 8 (37) | 1-2 : 5 |
|  |  | 3-8: 3 |
|  |  | 9-20:- |
|  |  | 21-40:- |
|  |  | >40:- |
| Nursing home physician   - Nursing home physicians without labeled hours - Nursing home physicians with labeled hours | 18 (32) | |
|  | - 6 (40) | |
|  | - 9 (60) | 1-2 : 5 |
|  |  | 3-8: 2 |
|  |  | 9-20:2 |
|  |  | 21-40: - |
|  |  | >40:- |
| General practitioner   - General practitioner s without labeled hours - General practitioners with labeled hours | 19 (33) | |
|  | - 6 (38) | |
|  | - 10 (62) | 1-2 : 8 |
|  |  | 3-8: 2 |
|  |  | 9-20:- |
|  |  | 21-40:- |
|  |  | >40:- |
| Spiritual counselor   - Spiritual counselors without labeled hours - Spiritual counselors with labeled hours | 37 (65) | |
|  | - 21 (64) | |
|  | - 12 (36) | 1-2 : 9 |
|  |  | 3-8: 2 |
|  |  | 9-20: 1 |
|  |  | 21-40:- |
|  |  | >40:- |
| Psychologist   - Psychologists without labeled hours - Psychologists with labeled hours | 16 (28) | |
|  | - 14 (93) | |
|  | - 1 (7) | 1-2 : 1 |
|  |  | 3-8: - |
|  |  | 9-20:- |
|  |  | 21-40:- |
|  |  | >40:- |
| Social worker   - Social workers without labeled hours - Social workers with labeled hours | 17 (28) | |
|  | - 14 (82) | |
|  | - 3 (18) | 1-2 : 2 |
|  |  | 3-4: 1 |
|  |  | 9-20: - |
|  |  | 21-40: - |
|  |  | >40: - |
| Mean number of disciplines participating in the teams (sd) | 6,5 (2,4) | |

*nurses include oncology nurses, pain nurses, palliative care nurses
